# Supplementary material for: What Works in Community-Led Suicide Prevention: Perspectives of Wesley LifeForce Network Coordinators
Source: Int J Environ Res Public Health. 2021 Jun 4;18(11):6084. doi: 10.3390/ijerph18116084 (PMC8200208; doi:10.3390/ijerph18116084)
Supplement: Supplementary file 1 [file ijerph-18-06084-s001.zip › ijerph-1228517-supplementary.pdf]

Supplementary Material S1: Survey tool

*** Introduction page ***

Thank you for participating in this survey.

<PLS and Consent form>

*** Part 1: Information about the Network and the person completing this survey ***

First, we are going to ask you some questions about your Network.

Q1: What is the name of your Network?

Q2: Where is your Network based?

State (dropdown)

Q3 What is the postcode of your Network location?

Postcode: ___ ___ ___ ___

Q4: In what year did the Network receive Wesley seed-funding?

Year: ___ ___ ___ ___

Don't know ❑

Q5: Since inception, has the Network received any additional funding, other than the seed funding from Wesley?

- ❑ Yes → go to Q6
- ❑ No → go to Q7
- ❑ Don't know → go to Q7

Q6: If yes, how much additional funding was received?

- ❑ 1 to 5,000 AUD
- ❑ 5,001 to 10,000 AUD
- ❑ 10,001 to 20,000 AUD
- ❑ Over 20,000 AUD

Q7: What type of funding sources? *(Please tick all applicable options.)*

- ❑ Donations
- ❑ Grants (such as Rotary or Beyond Blue)
- ❑ State/federal funding (including PHN funding)
- ❑ Fundraising events or activities
- ❑ Other (please specify) ______________

Q8: Approximately, how many individual members does the Network currently have?

Number: ___ ___

Q9: How many organisations, services or community groups are members of your Network?

Number: ___ ___

Q10: How many of the individual Network members are volunteers?

Number: ___ ___

Q11: How many of the individual Network members participate in the Network as part of their professional role/employment?

Number: ___ ___

Q12: To your knowledge, do any of the Network members have a lived experience of suicide?

By lived experience we mean people who have experienced suicidal thoughts, survived a suicide attempt, cared for someone going through a suicidal crisis, or have lost someone to suicide.

- ❑ Yes → go to Q12a
- ❑ No → go to Q13
- ❑ Don't know → go to Q13

Q12a: Approximately, how many Network members have a lived experience of suicide?

Number: ______ ❑ Don't know

Q13: What is the primary focus of your Network? *(please select only one)*

- ❑ General
- ❑ Youth
- ❑ LGBTIQ
- ❑ Indigenous
- ❑ CALD
- ❑ Industry
- ❑ Other, specify _____________

Q13a: Does you Network have another / secondary focus *(If so, please briefly describe below)*

Q14: Approximately, how many times has your Network met over the past 12 months? *(If you have not met in the last 12 months, enter '0')*

Number: ___ ___

The next questions are about you and your role in the Network.

Q15: What is your role in the Network?

- ❑ Chair/President
- ❑ Secretary
- ❑ Treasurer
- ❑ Project officer
- ❑ Member
- ❑ Other, specify___________________

Q16: Is your participation in the Network part of your professional role/employment or are you involved on a volunteer basis?

- ❑ Part of my professional job → go to Q17
- ❑ Involved on a voluntary basis → go to Q18
- ❑ Both as part of my professional job and as a volunteer → go to Q17

Q17: Please describe your professional role/employment of relevance to the Network.

____

Q18: How many hours on average do you typically contribute to the Network per week?

Average Hours (per week): ___ ___

Q19: Do you identify as having a lived experience of suicide?

By lived experience we mean people who have experienced suicidal thoughts, survived a suicide attempt, cared for someone going through a suicidal crisis, or have lost someone to suicide.

- ❑ Yes → go to Q20
- ❑ No → go to Q21
- ❑ Would rather not say → go to Q21

Q20: In what way do you identify as having a lived experience of suicide? *(please tick all that apply)*

- ❑ I have experienced suicidal thoughts
- ❑ I have survived a suicide attempt
- ❑ I have cared for someone through suicidal crisis
- ❑ I am bereaved by suicide

Q21: How long have you been involved with this Network? *(please move the slider)*

Number of Years (slider)

Q22: What has motivated you to join the Network? *(Please choose the main reason)*

- ❑ My lived experience of suicide
- ❑ I saw a need in the community and wanted to help create change
- ❑ My professional interest
- ❑ Other, please specify: ________________________

Q23: What is your age group?

- ❑ 18-19 years
- ❑ 20 to 29 years
- ❑ 30 to 39 years
- ❑ 40 to 49 years
- ❑ 50 to 59 years
- ❑ 60 to 69 years
- ❑ 70 to 79 years
- ❑ 80+ years

Q24: Do you identify as… ?

- ❑ Male
- ❑ Female
- ❑ Transgender male/female
- ❑ Non-binary/gender queer
- ❑ Different identity not listed here
- ❑ Prefer not to say

Q25: Are you of Aboriginal or Torres Strait Islander origin?

- ❑ No
- ❑ Yes, Aboriginal
- ❑ Yes, Torres Strait Islander
- ❑ Yes, both

* Part 2: Internal Network processes and outcomes *

The following questions are about the <u>internal processes and operations</u> of your Network. These are things the Network does internally rather than what they are doing externally with the community. We are interested in the internal activities of the Network and any outcomes of these.

Q26: Which of the following <u>internal processes and operations</u> has your Network done in either the last 12 months and/or more than 12 months ago?

If you are uncertain, or have not focused on these aspects before, please choose "Never" or "Not sure".

	In the past 12 months	More than 12 months ago	Never	Not sure
a. Identifying suicide prevention issues in the community	❑	❑	❑	❑
b. Identifying available suicide prevention frameworks	❑	❑	❑	❑
c. Identifying relevant community stakeholders	❑	❑	❑	❑
d. Identifying gaps in community knowledge on suicide	❑	❑	❑	❑
e. Identifying local service arrangements and referral pathways	❑	❑	❑	❑
f. Identifying local service needs and gaps, and existing barriers to service access and help-seeking	❑	❑	❑	❑
g. Identifying gaps in suicide prevention efforts	❑	❑	❑	❑
h. Training and capacity building of Network members	❑	❑	❑	❑
i. Participating in strategic planning to determine future Network directions and activities	❑	❑	❑	❑

Q27: Apart from those listed above, are there any other <u>internal processes and operations</u> that your Network is or has been doing? Please briefly list them here:

Open text: ____

The following statements refer to <u>potential outcomes</u> of your internal Network processes and operations for Network members.

Q28: As an outcome of our internal Network processes and operations, Network members now have a better <u>understanding</u> of the following:

Please indicate to what extent you agree to the statements below.

	Strongly disagree (1)	Dis-agree (2)	Neutral (3)	Agree (4)	Strongly agree (5)
a. Key suicide prevention issues in the community	❑	❑	❑	❑	❑
b. Existing suicide prevention frameworks	❑	❑	❑	❑	❑
c. Local service arrangements and referral pathways	❑	❑	❑	❑	❑
d. Gaps in community knowledge regarding suicide	❑	❑	❑	❑	❑
e. Local service needs and gaps	❑	❑	❑	❑	❑
f. Gaps in suicide prevention efforts	❑	❑	❑	❑	❑
g. Help-seeking barriers and facilitators	❑	❑	❑	❑	❑
h. Service access barriers and facilitators	❑	❑	❑	❑	❑

Q29: As an outcome of our internal Network processes and operations, Network members now have increased <u>confidence and capacity</u> to do the following:

Please indicate to what extent you agree to the statements below.

	Strongly disagree (1)	Dis-agree (2)	Neutral (3)	Agree (4)	Strongly agree (5)
a. Collaboratively plan and develop network-initiated strategies	❑	❑	❑	❑	❑
b. Plan initiatives to address knowledge gaps	❑	❑	❑	❑	❑
c. Plan initiatives to address service gaps and access issues	❑	❑	❑	❑	❑
d. Plan initiatives to encourage help-seeking	❑	❑	❑	❑	❑
e. Plan initiatives to strengthen community responses	❑	❑	❑	❑	❑
f. Plan initiatives to enhance service responses	❑	❑	❑	❑	❑
g. Evaluate network initiatives	❑	❑	❑	❑	❑

*** Part 3: External Network activities and outcomes ***

The following questions are about the community focused activities and outcomes of your Network. These are things the Network does externally with the community.

Q30: Which of the following community focused activities has your Network done in either the last 12 months and/or more than 12 months ago?

If you are uncertain, or have not focused on these aspects before, please choose "Never" or "Not sure".

	In the past 12 months	More than 12 months ago	Never	Not sure
a. Distributing local support service contact information	❑	❑	❑	❑
b. Facilitating community suicide awareness and stigma reduction initiatives	❑	❑	❑	❑
c. Initiating suicide prevention activities that address needs of diverse populations and service gaps	❑	❑	❑	❑
d. Facilitating bereavement support and postvention activities	❑	❑	❑	❑
e. Fostering recognition and capacity of lived experience in suicide prevention	❑	❑	❑	❑
f. Facilitating access to suicide prevention training	❑	❑	❑	❑
g. Supporting services to build their capacity in identifying, responding to and assisting suicidal people	❑	❑	❑	❑
h. Facilitating community access to support services	❑	❑	❑	❑
i. Advocating and promoting responsive service policies, proactive guidelines and appropriate referral pathways	❑	❑	❑	❑

Q31: Apart from those listed above, are there any other key community focused activities your Network is or has been doing? Please briefly list them here:

Open text: ____

The following statements refer to potential outcomes of your community focused activities.

Q32: As an outcome of the Network's community focused activities, the <u>community</u> now has:

Please indicate to what extent you agree to the statements below.

	Strongly disagree (1)	Dis-agree (2)	Neutral (3)	Agree (4)	Strongly agree (5)
a. Increased knowledge about support services and their linkages	❑	❑	❑	❑	❑
b. Improved awareness of suicide prevention services/strategies	❑	❑	❑	❑	❑
c. Increased confidence in assisting people at risk of suicide	❑	❑	❑	❑	❑
d. Increased capacity to respond and help someone at risk of suicide	❑	❑	❑	❑	❑

Q33: As an outcome of the Network's community focused activities, <u>service providers</u> now have:

Please indicate to what extent you agree to the statements below.

	Strongly disagree (1)	Dis-agree (2)	Neutral (3)	Agree (4)	Strongly agree (5)
a. Improved awareness of suicide prevention services/strategies	❑	❑	❑	❑	❑
b. Increased confidence in assisting people at risk of suicide	❑	❑	❑	❑	❑
c. Increased capacity to respond and help someone at risk of suicide	❑	❑	❑	❑	❑
d. Included people with lived experience in suicide prevention activities	❑	❑	❑	❑	❑
e. Improved service linkages and access pathways	❑	❑	❑	❑	❑

Q34: As an outcome of the Network's community focused activities, <u>people at risk of suicide</u>:

Please indicate to what extent you agree to the statements below.

	Strongly disagree (1)	Dis-agree (2)	Neutral (3)	Agr ee (4)	Strongly agree (5)
a. Experience less stigma in the community	❑	❑	❑	❑	❑
b. Are identified early	❑	❑	❑	❑	❑
c. Receive appropriate support and referral to relevant support services	❑	❑	❑	❑	❑
d. Demonstrate increased help-seeking behaviour and uptake of available services	❑	❑	❑	❑	❑
e. Are supported by people in the community	❑	❑	❑	❑	❑

Q35: Overall, since the Network began, we have seen the following outcomes:

Please indicate to what extent you agree to the statements below.

	Strongly disagree (1)	Dis-agree (2)	Neutral (3)	Agr ee (4)	Strongly agree (5)
a. Suicide risk in the community has been reduced	❑	❑	❑	❑	❑
b. The coordination of suicide prevention efforts has improved	❑	❑	❑	❑	❑
c. Suicide prevention efforts follow a whole of community approach	❑	❑	❑	❑	❑
d. Community wellbeing has improved	❑	❑	❑	❑	❑

*** Part 4: And a few last questions ***

In this final section we would like to learn more about what factors helped the Network to achieve its outcomes and if there were any barriers to effective network functioning.

Q36: What were the key factors (if any) that helped your Network function efficiently and achieve its outcomes? Please briefly describe the main three facilitators.

Open text: ____

Q37: What were the key barriers (if any) that inhibited the Network from functioning efficiently and achieve its outcomes? Please briefly describe the main three barriers.

Open text: ____

Q38: For the future, what is needed for the Network to continue to work successfully?

Open text: ____

Q39: Has being part of this Network made a difference to your life?

- ❑ Yes → go to Q40
- ❑ No → go to Q41
- ❑ Don't know → go to Q41

Q40: If yes, can you briefly describe what has changed for you.

Open text: ____

Q41: Is there anything else that you would like to share with us?

For example, have we missed any other outcomes that you have observed in your community; or were there any unintended outcomes or unforeseen consequences (positive or negative) of the Network initiatives?

Open text: ____

Thank you very much for completing this survey. The information you have provided will be very useful in understanding the breadth and depth of Wesley Network activities and outcomes.

❑ Submit survey

Administration notes: Last page will show the confirmation of survey submission and list contact details of project team.

Supplementary Material S2: Profiles of invited, responding and non-responding networks

	Invited (N=92)		Responding (N=41)		Nonresponding (N=51)	
**Status**	n	%	n	%	n	%
**Incubating**	10	10.87	4	9.76	6	11.76
**Sustainable**	82	89.13	37	90.24	45	88.24
**Focus**						
**Indigenous**	19	20.65	5	12.20	14	27.45
**General**	65	70.65	34	82.93	34	66.67
**Other**	8	8.70	2	4.88	3	5.88
**State**						
**NSW**	28	30.43	12	29.27	16	31.37
**Qld**	21	22.83	9	21.95	12	23.53
**Vic**	14	15.22	9	21.95	5	9.80
**WA**	10	10.87	4	9.76	6	11.76
**SA**	8	8.70	1	2.44	7	13.73
**NT**	7	7.61	3	7.32	4	7.84
**Tas**	3	3.26	3	7.32	0	0.00
**ACT**	1	1.09	0	0.00	1	1.96
**Rurality**						
**Regional**	42	45.65	18	43.90	24	47.06
**Major city**	28	30.43	13	31.71	15	29.41
**Remote**	22	23.91	10	24.39	12	23.53

Note. Of 47 unique survey responses received (response rate 51.1%), 41 contained sufficient data to be included in analysis (response rate 44.6%).
